# Association of DNA Methyltransferases 3A and 3B Polymorphisms, and Plasma Folate Levels with the Risk of Urothelial Carcinoma

**DOI:** 10.1371/journal.pone.0104968

**Published:** 2014-08-15

**Authors:** Chi-Jung Chung, Chao-Hsiang Chang, Chiu-Shong Liu, Chi-Ping Huang, Yi-Huei Chang, Ssu-Ning Chien, Ping-Huan Tsai, Hui-An Hsieh

**Affiliations:** 1 Department of Health Risk Management, College of Public Health, China Medical University, Taichung, Taiwan; 2 Department of Medical Research, China Medical University Hospital, Taichung, Taiwan; 3 Department of Urology, China Medical University and Hospital, Taichung, Taiwan; 4 Department of Medicine, College of Medicine, China Medical University and Hospital, Taichung, Taiwan; 5 Department of Family Medicine, China Medical University, Taichung, Taiwan; Tulane University Health Sciences Center, United States of America

## Abstract

**Background:**

Interindividual genetic variations of human DNA methyltransferases (DNMTs), which involve the methyl donor from the folate-related one-carbon metabolism pathway, are hypothesized as a risk factor for urothelial carcinoma (UC). Therefore, we evaluated the role of gene-environment interaction in UC carcinogenesis.

**Methods:**

A hospital-based case-control study was conducted by recruiting 192 patients with UC and 381 controls. Their plasma folate levels were measured using a competitive immunoassay kit. In addition, DNMT3A −448A>G and DNMT3B −579G>T genotyping was evaluated using a polymerase chain reaction-restriction fragment length polymorphism technique. Multivariate logistic regression and 95% confidence intervals (CIs) were applied to estimate the UC risk.

**Results:**

We observed that patients with UC exhibited a higher prevalence rate of folate insufficiency (folate levels ≤6 ng/mL) compared with the controls (35.94% and 18.37%, respectively). Furthermore, folate levels were higher in the prevalent UC patients than in the incident UC patients. However, folate insufficiency was similarly associated with a nearly two-fold increase in the risk of UC regardless of the UC patient group. In addition, the frequencies of the variant alleles for DNMT3A and DNMT3B were 0.80 and 0.92, respectively, and no association was observed with UC risk. However, participants with a variant homozygous genotype of DNMT3B −579G>T and folate insufficiency or with high cumulative cigarette smoking exhibited an increased risk of UC.

**Conclusion:**

Overall, environmental factors may contribute more significantly to UC carcinogenesis compared with genetic susceptibility. Future studies should investigate other polymorphisms of DNMT3A and DNMT3B to determine genetic susceptibility.

## Introduction

Bladder cancer is the most common type of urothelial carcinoma (UC), and, based on the primary anatomical site, it currently ranks among the top 10 frequent cancers in Taiwan, particularly for men older than 40 y (Department of Health, the Executive Yuan). Although the mechanism of UC carcinogenesis is poorly understood, exposure to cigarette smoke is one of the most critical risk factors for bladder cancer, accounting for up to 50% of all new cases [1]. Previous studies have indicated that several carcinogens in cigarette-related products, such as nicotine-derived, tobacco-specific nitrosamines and aromatic amines, enter the human body, and their specific metabolic forms cumulate in the bladder and are finally excreted in the urine [2]–[4]. Although the mechanism by which cigarette smoking increases the risk of UC remains unclear, the free radicals produced from the metabolism of these carcinogens may directly or indirectly induce oxidative damage of the bladder epithelium; this indicates an association between smoking and the risk of UC [5], [6]. In addition, aside from the effects of cigarette smoking on UC risk, studies have explored the effects of the one-carbon metabolism pathway and DNA methylation on UC carcinogenesis.

DNA methylation, one of the most commonly studied epigenetic phenomena, is a natural postgenomic modification that requires adding a methyl group to the 5′ position of the cytosine ring in the CpG dinucleotides to form 5-methylcytosine (5-MeC) [7]. In addition, S-adenosylmethionine (SAM), the unique methyl donor involved in DNA methylation, is derived from the folate and methionine cycles [8]. Folate (vitamin B9) is an initial methyl donor in methionine biosynthesis and is required for crucial cell processes [9], [10]. Folate deficiency causes several metabolic changes in the cell, including hyperhomocysteinemia, low SAM levels, and DNA hypomethylation [11]. According to the Nutrition and Health Survey in Taiwan (NAHSIT) 2005−2008, the prevalence of folate insufficiency (≤6 ng/mL) in men was higher than that in women (34.1% and 14.8%, respectively) [12]. Most previous studies have reported that individuals with folate deficiency or hyperhomocysteinemia exhibit an increased risk of UC [13], [14].

DNA methyltransferases (DNMTs) are enzymes responsible for maintaining the methylation patterns [7]. Previous literature indicates that DNA methylation profiles, including the 5-MeC and DNMT1 levels, regulate the epigenetic control of gene transcription, affect tissue-specific gene expression, and are associated with various biological processes including carcinogenesis [7], [8]. However, the differential susceptibility may be attributed to polymorphisms in genes that encode the DNA methylation-related enzymes, including DNMT3A −448A>G (rs1550117) and DNMT3B −579G>T (rs1569686), which are the most widely studied single nucleotide polymorphisms (SNPs). Increasing evidence from epidemiological studies suggests an association between the SNPs of DNMT3A and DNMT3B [15]–[17]. However, the results remain controversial, depending on the varied ethnicity, tumor types, and study designs.

Based on relevant literature, plasma folate insufficiency and genetic polymorphisms of DNMT3A and 3B might affect the cellular DNA methylation levels [10]. In addition, recent studies have indicated that cigarette smoke may modify DNA methylation through the effects of nicotine on the DNMT mRNA gene expression [18]. Although previous research has reported the significant effects of plasma folate levels or exposure to cigarette smoke on UC risk, few studies have investigated the prevalence of genetic polymorphisms of DNMT3A and DNMT3B in Taiwan or the interactions among cigarette smoke and plasma folate, stratified by DNMT3 polymorphism, and their effects on the risk of UC. Therefore, we conducted a hospital-based case-control study to evaluate the association of DNMT3A and DNMT3B gene polymorphisms, plasma folate levels, and exposure to cigarette smoke with the risk of UC.

## Methods

### Study participants

We conducted a hospital-based case-control study and enrolled 192 patients with UC and 381 controls from June 2011 to December 2013. All of the study participants were recruited from the China Medical University Hospital. Patients with UC comprised outpatients or inpatients at the Department of Urology and included the incident and prevalent cases diagnosed among men and women aged 30−90 y; the UC cases were limited to patients with urinary tract urothelial carcinoma, whose diagnoses were evaluated by a pathologist. In addition, we distinguished the prevalent and incident UC cases by using the date of operation, pathological diagnosis, and recruitment, as well as the self-report from patients. The control participants were recruited from among individuals receiving adult health examinations at the Department of Family Medicine and elected through frequency matching with cases according to sex and age category (every 5 years each). Finally, 192 UC cases, including 104 incident cases and 88 prevalent cases, and 381 controls were included in the analysis. The mean prevalent duration of the 88 UC cases was 3.08 y (min–max: 0.08−12.90 y). All study participants provided informed consent before questionnaire interviews and blood sample collection. The Research Ethics Committee of the China Medical University Hospital in Taichung, Taiwan approved the study (DMR100-IRB-080 and DMR100-IRB-262), and the study protocol was performed in accordance with the World Medical Association Declaration of Helsinki.

### Questionnaire interview

Structural questionnaires were administered through face-to-face interviews, and the study participants were requested to provide detailed information regarding demographics, socioeconomic characteristics, lifestyle factors (such as cigarette smoking and environmental exposure to smoke), as well as personal and family medical history.

### Biological specimen collection

During the physical examinations, we used ethylenediaminetetraacetic acid (EDTA)-vacuumed syringes to collect 5−8 mL of peripheral blood samples, which were centrifuged at 3,000 ×g for 10 min to separate the buffy coat and the plasma and then frozen at −20°C to measure the plasma folate and DNA extraction levels.

### Plasma folate determination

The plasma folate levels were measured using a competitive immunoassay kit (ADVIA Centaur Folate assay, Siemens) by using the direct chemiluminescent technology according to the manufacturer’s instructions. All plasma samples were evaluated under dim yellow light. For replicate plasma samples, the mean coefficient of variation was <10%.

### DNA extraction and genotyping of SNPs

Following phenol and chloroform extraction, genomic DNA was extracted from peripheral blood mononuclear cells by using proteinase K digestion. In brief, cells were lysed using a cell lysis solution, and then, the RNA in the sample was digested using an RNase A solution. The protein was precipitated using a protein precipitation solution. Finally, isopropanol was used to precipitate the genomic DNA, followed by washing with 70% ethanol. The SNPs in DNMT3A −448A>G (rs1550117) and DNMT3B −579G>T (rs1569686) were genotyped using a polymerase chain reaction (PCR)-restriction fragment length polymorphism method [15], [19]. The following primers were used to amplify the 358 bp and 225 bp PCR products: 5′- ACACACCGCCCTCACCCCTT-3′ (forward) and 5′- TGTGGGCAGGGATTGCTGGA-3′(reverse) for DNMT3A; and 5′-GAGGTCTCATTATGCCTAGG-3′ (forward) and 5′-GGGAGCTCACCTTCTAGAAA-3′ (reverse) for DNMT3B. A total of 30 µL of PCR products was obtained, which comprised 80 ng of sample DNA, 10× PCR buffer, 2.5 mM dNTP, 2 µM each primer, and 1 U of Taq polymerase. After initial denaturation for 4 min at 94°C, 35 cycles were performed at 94°C for 40 s (denaturation), at 66.4°C for 30 s (annealing), and at 72°C for 30 s (extension) each for DNMT3A and at 94°C for 30 s, at 56°C for 30 s, and at 72°C for 30 s each for DNMT3B, followed by a final step at 72°C for 5 min. The amplified products were visualized by electrophoresis in 2% agarose gels. The PCR products were digested with TaaI (for 1 h at 65°C) for DNMT3A and with PvuII (for 1 h at 37°C) for DNMT3B. The products were analyzed by electrophoresis on 3% agarose gels. Approximately 5% of the samples were randomly extracted and repeated with 100% concordance for quality control.

### Statistical analysis

The genotype frequencies in the controls, as expected under the Hardy-Weinberg equilibrium, were tested for goodness of fit by using the χ^2^ test. In addition, the SNPs of DNMT3A −448A>G and DNMT3B −579G>T were divided into three classes, namely, wild-type homozygotes, variant heterozygotes, and variant homozygotes. Cigarette smoking status included never, former, and current. Former smokers were defined as those who had quit cigarette smoking, and current smokers were those who were still smoking at the time of recruitment. Cumulative cigarette smoking (pack-years) was derived by summing the number of years of smoking and the average amount smoked daily during that period. Moreover, the cutoff points of cigarette smoking-related variables were determined by the median value among the controls. According to the findings of Chen et al. based on the surveys of NAHSIT in Taiwan, folate levels <3 ng/mL (6.8 nmol/L) and ≤6 ng/mL (13.5 nmol/L) indicated folate deficiency and folate insufficiency, respectively [12]. In addition, we adopted the tertile or quartile cutoff points determined from the plasma folate levels among the controls to evaluate the association between folate levels and UC risk. Nonparametric analysis was applied to compare the differences of plasma folate levels between the UC cases and controls or between the incident and prevalent UC cases. Simple and multivariate logistic regression models were used to estimate the odds ratios (ORs) and 95% confidence intervals (CIs) to determine the association between the DNMT3A and DNMT3B genotypes with the risk of UC after adjustment for age and sex or other potential factors. Finally, we used the multiplicative model to evaluate the combined effects of plasma folate levels and gene polymorphism on the risk of UC. All analyses were conducted using the Statistical Analysis Software (SAS) statistical package (SAS, version 8.0, Cary, NC, USA).

## Results

### Characteristics, cigarette smoking, and UC risk

Table 1 lists the frequencies of sociodemographic and clinical characteristics as well as the cigarette smoking status. The mean age of all participants at recruitment was approximately 66 y. Approximately half of participants (53%) were male. Healthy controls attained higher educational levels than did patients with UC. On average, more than half of the patients with UC were nonsmokers. Based on multivariate logistic regression models after adjustment for age and gender, the ORs for UC were 3.39 (95% CI, 1.97−5.84) and 2.69 (95% CI, 1.40−5.14) in participants who were former and current smokers, respectively, compared with those who were nonsmokers. Furthermore, we stratified the participants based on several cigarette smoking-related variables, including duration of cigarette smoking, amount of cigarette smoking, and cumulative cigarette smoking. We observed a significant dose-response relationship for patients with higher levels of cigarette smoking after adjustment for age and sex (trend *P*<0.05). In other words, participants with longer duration or higher amount of cigarette smoking or cumulative cigarette smoking exhibited a significantly increased risk of UC.

**Table 1:** Sociodemographic and clinical characteristics, as well as cigarette smoking habits of 192 UC patients and 381 matched controls.

	UC patients (n = 192)	Controls (n = 381)	OR^a^ (95% CI)
Condition of UC			
Incidence cases	104		
Prevalence cases	88 (duration: 0.08–12.90; mean: 3.08 years)		
Age (years)	67.37±0.77	66.17±0.52	
Male (%)	102 (53.13)	203 (53.28)	
BMI	23.68±0.24	24.29±0.16	0.94 (0.89–0.99)
Educational level (%)			
Elementary school	109 (56.77)	98 (25.72)	1.00*
Junior high school	57 (29.69)	164 (43.04)	0.29 (0.19–0.45)
College or above	26 (13.54)	119 (31.23)	0.18 (0.11–0.30)
Cigarette smoking habits			
Non-smoker	118 (61.46)	289 (75.85)	1.00*
Former smoker	50 (26.04)	53 (14.17)	3.39 (1.97–5.84)
Current smoker	24 (12.50)	38 (9.97)	2.69 (1.40–5.14)
Ever smokers	74 (38.54)	92 (24.15)	3.03 (1.86–4.94)
Duration of cigarette smoking (years)	14.57±1.45	7.58±0.79	1.03 (1.02–1.05)
0	118 (61.78)	289 (76.46)	1.00*
<32.1	24 (12.57)	47 (12.43)	1.96 (1.05–3.65)
≥32.1	49 (25.65)	42 (11.11)	4.33 (2.46–7.64)
Missing data	0	4	
Amount of cigarette smoking (pack/day)	0.37±0.04	0.21±0.02	2.22 (1.50–3.28)
0	118 (61.78)	289 (75.85)	1.00*
<1.1	30 (15.71)	45 (11.81)	2.47 (1.37–4.48)
≥1.1	43 (22.51)	47 (12.34)	3.55 (2.08–6.26)
Missing	1	0	
Cumulative cigarette smoking (pack-years)	13.96±1.56	7.29±0.93	1.02 (1.01–1.03)
0	118 (62.11)	289 (76.46)	1.00*
0–26.76	24 (12.63)	45 (11.90)	2.02 (1.09–3.75)
≥26.76	48 (25.26)	44 (11.64)	4.14 (2.35–7.28)
Missing data	1	4	

a OR values were evaluated by using multivariate logistic regression model after adjustment for age and gender.

*trend *p*<0.05.

### Association between plasma folate and UC risk

The differences in plasma folate levels were compared between the patients with UC and the controls (Table 2). Patients with UC exhibited slightly lower plasma folate levels than those of the controls; however, the Wilcoxon rank-sum test revealed no significant statistical difference between the folate levels for both groups. However, participants with folate insufficiency (≤6 ng/mL) exhibited a twofold increase in the risk of UC than did those without folate insufficiency (>6 ng/mL) after we adjusted for other risk factors. In addition, similar results were observed in participants with folate deficiency (<3 ng/mL). After adjusting for age, sex, education, and cumulative cigarette smoking, we observed a 3.08-fold risk of UC (95% CI: 1.20−7.85) in participants with folate deficiency compared with those without folate deficiency (data not shown). Moreover, after we adjusted for potential confounders, participants with higher plasma folate levels revealed a significantly decreased risk of UC, regardless of the tertile or quartile cutoff point of plasma folate levels used for analysis (trend *P*<0.05). Furthermore, we compared the differences in folate levels among the incident (n = 104) and prevalent (n = 88) UC cases; the prevalent UC cases revealed slightly higher plasma folate levels than the incident UC cases (median ± SD: 8.45±2.17 vs. 7.28±1.33, respectively). Particularly incident UC cases with higher plasma folate levels exhibited a significantly decreased risk of UC in the multivariate models. However, this was not observed in the prevalent UC cases after adjustment for other risk factors. Nevertheless, regardless of the patient group, the association between folate insufficiency or deficiency and the risk of UC was similar in all analyses.

**Table 2:** Associations between the levels of plasma folate and UC risk by using multivariate logistic regression models.

Plasma folate (ng/mL)	UC patients N = 192	Controls N = 381	OR^a^ (95% CI)	OR^b^ (95% CI)
Median ± S.D.	7.73±1.23	9.82±0.37	1.01 (0.99–1.03)	1.02 (0.99–1.04)
>6	123 (64.06)	311 (81.63)	*ref.*	*ref.*
≤6	69 (35.94)	70 (18.37)	2.57 (1.73–3.84)	2.06 (1.34–3.15)
<7.88	101 (52.60)	127 (33.33)	*ref.* *	*ref.* *
7.88–13.17	44 (22.92)	128 (33.60)	0.42 (0.27–0.65)	0.48 (0.30–0.76)
≥13.17	47 (24.48)	126 (33.07)	0.44 (0.28–0.68)	0.61 (0.38–0.97)
<6.75	83 (43.23)	97 (25.46)	*ref.* *	*ref.^#^*
6.75–9.83	33 (17.19)	94 (24.67)	0.41 (0.26–0.68)	0.46 (0.27–0.77)
9.83–14.9	37 (19.27)	98 (25.72)	0.42 (0.26–0.68)	0.49 (0.29–0.83)
≥14.9	39 (20.31)	92 (24.15)	0.46 (0.28–0.75)	0.66 (0.39–1.11)
Incidence cases	N = 104	N = 381		
Median ± S.D.	7.28±1.33^c^	9.82±0.37	0.99 (0.97–1.02)	1.01 (0.99–1.04)
>6	123 (64.06)	311 (81.63)	*ref.*	*ref.*
≤6	69 (35.94)	70 (18.37)	3.09 (1.90–5.01)	2.43 (1.45–4.06)
<7.88	101 (52.60)	127 (33.33)	*ref.* *	*ref.* *
7.88–13.17	44 (22.92)	128 (33.60)	0.34 (0.19–0.61)	0.40 (0.22–0.73)
≥13.17	47 (24.48)	126 (33.07)	0.37 (0.22–0.65)	0.53 (0.30–0.95)
<6.75	83 (43.23)	97 (25.46)	*ref.* *	*ref.* *
6.75–9.83	33 (17.19)	94 (24.67)	0.38 (0.21–0.71)	0.44 (0.23–0.84)
9.83–14.9	37 (19.27)	98 (25.72)	0.33 (0.18–0.62)	0.39 (0.20–0.76)
≥14.9	39 (20.31)	92 (24.15)	0.37 (0.20–0.68)	0.53 (0.28–1.03)
Prevalence cases	N = 88	N = 381		
Median ± S.D.	8.45±2.17^c^	9.82±0.37	1.03 (0.99–1.05)	1.03 (0.99–1.05)
>6	123 (64.06)	311 (81.63)	*ref.*	*ref.*
≤6	69 (35.94)	70 (18.37)	2.11 (1.24–3.60)	1.76 (1.01–3.08)
<7.88	101 (52.60)	127 (33.33)	*ref.* *	*ref.*
7.88–13.17	44 (22.92)	128 (33.60)	0.52 (0.29–0.92)	0.56 (0.31–1.01)
≥13.17	47 (24.48)	126 (33.07)	0.50 (0.27–0.90)	0.67 (0.36–1.25)
<6.75	83 (43.23)	97 (25.46)	*ref.^#^*	*ref.*
6.75–9.83	33 (17.19)	94 (24.67)	0.44 (0.22–1.00)	0.45 (0.23–0.91)
9.83–14.9	37 (19.27)	98 (25.72)	0.53 (0.28–1.00)	0.60 (0.31–1.18)
≥14.9	39 (20.31)	92 (24.15)	0.56 (0.29–1.08)	0.76 (0.38–1.51)

a OR values were adjusted for age and gender.

b OR values were adjusted for age, gender, educational level, and cumulative cigarette smoking.

c *p* = 0.1772 by Student *t*-test.

*p<0.05 by trend test; *^#^*0.1<*p*<0.05 by trend test.

### Gene polymorphisms of DNMT3A and DNMT3B and UC risk

Table 3 summarizes the distribution of DNMT3A and DNMT3B genotypes and the individual ORs of UC. All genotype frequencies of DNMT were fitted using the Hardy-Weinberg equilibrium. The frequencies of the variant alleles for DNMT3A and DNMT3B were 0.80 and 0.92, respectively. Simple logistic regression analysis revealed that for DNMT3A −448A>G, the participants with either a heterozygous (OR 1.09; 95% CI = 0.36−3.26, *P* = 0.88) or a variant homozygous (OR 1.32; 95% CI = 0.45−3.83, *P* = 0.61) genotype exhibited a positive association with the risk of UC, in contrast to those with a wild-type homozygous genotype. Moreover, for DNMT3B −579G>T, when the participants with wild-type homozygous or heterozygous genotypes were combined as the reference group, those with the variant homozygous genotype exhibited a positive association with the risk of UC compared with the reference group (OR 1.07; 95% CI = 0.63−1.81, *P* = 0.81). However, no statistical significance was observed in our analysis after adjustment for other risk factors.

**Table 3:** Odd ratios of UC risk were evaluated by using simple and multivariate logistic regression models by stratification of DNMT3A −448A>G and DNMT3B −579G>T genotypes.

	UC patients	Controls	OR (95% CI)	OR^a^ (95% CI)
Number	192	381		
*DNMT3A* −448A>G (rs1550117)				
WW	5 (2.60)	12 (3.15)	*ref.*	*ref.*
WV	48 (25.00)	106 (27.82)	1.09 (0.36–3.26)	0.81 (0.25–2.56)
VV	117 (60.94)	213 (55.91)	1.32 (0.45–3.83)	0.99 (0.32–3.05)
Missing data	22 (11.46)	50 (13.12)	1.06 (0.33–3.36)	0.82 (0.24–2.81)
Dominant (WV+VV vs. WW)			1.24 (0.43–3.58)	0.93 (0.31–2.84)
Recessive (VV vs. WW+WV)			1.22 (0.82–1.82)	1.21 (0.79–1.84)
*p* value for Hardy–Weinberg equilibrium = 0.79 (Var freq: 0.80)				
*DNMT3B* −579G>T (rs1569686)				
WW	0	3 (0.79)		
WV	24 (12.50)	48 (12.60)	*ref.*	*ref.*
VV	139 (72.40)	277 (72.70)	1.07 (0.63–1.81)	1.17 (0.66–2.05)
Missing	29 (15.10)	53 (13.91)	1.16 (0.60–2.26)	1.23 (0.61–2.51)
Dominant (WV+VV vs. WW)			–	–
Recessive (VV vs. WW+WV)			1.07 (0.63–1.81)	1.13 (0.65–1.98)
*p* value for Hardy–Weinberg equilibrium = 0.57 (Var freq: 0.92)				

W/W: wild-type homozygotes; W/V: heterozygotes; V/V: variant homozygotes.

a OR values were adjusted for age, gender, educational level, and cumulative cigarette smoking.

### Association of gene-environment interaction with UC risk

The Wilcoxon rank-sum test was used to analyze the differences in plasma folate levels in the DNMT3A −448A>G and DNMT3B −579G>T genotypes for the controls. Participants with the DNMT3A −448A>G heterozygous genotype or the variant homozygous genotype exhibited lower plasma folate levels than did those with the wild-type homozygous genotype (11.58±6.80 vs. 14.11±7.29 ng/mL; *P* = 0.06). Moreover, participants with the DNMT3B −579G>T variant homozygous genotype exhibited lower plasma folate levels than did those with the wild-type homozygous or the heterozygous genotype (11.60±7.18 vs. 13.14±8.45 ng/mL; *P* = 0.08). In addition, participants with high cumulative cigarette smoking exhibited low plasma folate levels based on the Spearman correlation analysis (*r* = –0.22, *P*<0.0001) (data not shown). Therefore, the relationship of plasma folate levels, cigarette smoking, and DNMT gene polymorphisms with the risk of UC were evaluated (Table 4). Furthermore, for DNMT3B −579G>T, participants with the variant homozygous genotype and lower plasma folate levels (≤6 ng/mL) or and with cumulative cigarette smoking (>0) exhibited a significantly increased risk of UC than did those with the heterozygous or the wild-type homozygous genotype and with higher plasma folate levels (>6 ng/mL) or and with no cumulative cigarette smoking (OR 2.27; 95% CI = 1.09−4.73, *P* = 0.81 vs. OR 2.63; 95% CI = 1.11−6.22, *P* = 0.81). However, the results were not similar for the combination of DNMT3A −448A>G genotype and plasma folate or cumulative cigarette smoking. Finally, in the multiplicative models, all interaction p values of plasma folate and cumulative cigarette smoking revealed no statistical significance for UC risk (all *P* values >0.05).

**Table 4:** Interaction between cigarette smoking and plasma folate stratified by *DNMT3* polymorphism on UC risk evaluated by multivariate logistic regression models.

		*DNMT3A* −448A>G (rs1550117)						
		WW *(n = 17)*					WV+VV *(n = 484)*	
		Case/Control				Adjusted OR^a^ (95% CI)	Case/Control	Adjusted OR^a^ (95% CI)
Plasma folate (ng/mL)	>6	5/12				*ref.*	107/259	0.81 (0.26–2.46)
	≤6	0/0				–	58/60	1.60 (0.50–5.14)
							Interaction p = 0.6104	
Cumulative cigarette smoking(pack-years)	= 0	4/11				*ref.*	101/240	0.93 (0.27–3.14)
	>0	1/1				5.58 (0.25–123.97)	63/77	2.03 (0.54–7.61)
							Interaction p = 0.5394	
Cumulative cigarette smoking(pack-years)	Plasma folate							
= 0	>6	4/11			*ref.*		71/203	0.90 (0.26–3.06)
	≤6	0/0			–		30/37	1.99 (0.54–7.34)
>0	>6	1/1			5.65 (0.25–125.63)		36/55	2.21 (0.58–8.45)
	≤6	0/0			–		27/22	3.51 (0.87–14.24)
		***DNMT3B*** ** −** ***579G>T (rs1569686)***						
		**WW+WV ** ***(n = 75)***					**VV ** ***(n = 416)***	
		**Case/Control**		**Adjusted OR** a **(95% CI)**			**Case/Control**	**Adjusted OR** a **(95% CI)**
Plasma folate (ng/mL)	>6	16/42		*ref.* *			91/226	1.20(0.63–2.30)
	≤6	8/9		2.65(0.79–8.86)			48/51	2.27(1.09–4.73)
							Interaction p = 0.6124	
Cumulative cigarette smoking(pack-years)	= 0	12/39		*ref.* *			91/210	1.47(0.72–3.02)
	>0	12/12		3.98(1.29–12.29)			46/65	2.63(1.11–6.22)
							Interaction p = 0.1820	
Cumulative cigarette smoking(pack-years)	Plasma folate							
= 0	>6	9/33	*ref.* *				63/179	0.97(0.36–2.56)
	≤6	3/6	–				28/31	2.06(0.66–6.42)
>0	>6	7/9	1.96(0.37–10.41)				28/46	1.91(0.58–6.34)
	≤6	3/5	6.14(0.75–50.50)				18/19	2.79(0.76–10.22)

*ref.*: reference group.

a Multivariate OR values were adjusted for age, gender, educational level, and cumulative cigarette smoking.

*p<0.05 by trend test.

## Discussion

Based on our research, this is the first study to evaluate simultaneously the relationships among the gene polymorphisms of DNMT3A or DNMT3B, plasma folate levels, cigarette smoking exposure, and UC risk. In the present study, participants with folate insufficiency exhibited a twofold increase in the risk of UC, regardless of whether they had prevalent or incident UC. Moreover, no association was observed between the DNMT genotype and UC risk. However, participants with a variant homozygous genotype of DNMT3B −579G>T and with folate insufficiency or high cumulative cigarette smoking exhibited an increased risk of UC.

Folate, a water-soluble B vitamin in green leafy vegetables, citrus fruits, and legumes, is the initial methyl donor in methionine biosynthesis [10]. Folate insufficiency disrupts the transfer of the one-carbon units involved in all biochemical reactions including plasma homocysteine determinants and SAM synthesis [11], [20]. A previous study investigated the 10-y trends in plasma folate levels in Taiwanese people through three national NAHSIT surveys conducted in 1993−1996, 1999−2000, and 2005−2008 and reported similar plasma folate levels of approximately 8 ng/mL in men and 11 ng/mL in women; however, the prevalence of folate deficiency (<3 ng/mL) increased during these 10-y periods, particularly for men [12]. In our study, the median value of plasma folate levels was similar to that reported by Chen et al. but was higher than that reported in other studies [12], [21], [22]. Moreover, in our study, the prevalence of folate insufficiency was higher than that in other studies, ranging from 5% to 15% [22]. The differences in the prevalence of folate insufficiency may be attributed to the ethnic variance of dietary habits or individual susceptibility to polymorphisms in metabolizing folate-related genes such as DNMT3A and 3B. Previous studies have indicated a negative association between plasma folate levels (or decreased folate intake) and the risks of various cancers [14], [23], [24]. These findings were consistent with our results, although other studies have presented contradictory findings [25], [26].

Folate is a precursor of SAM, the primary methyl group donor for most biological methylation reactions, that indirectly affects DNA methylation and epigenetic gene regulation, which is crucial to carcinogenesis [27]. The human DNMT family is primarily categorized into DNMT1, DNMT3A, and DNMT3B, which encode the maintenance and de novo methyltransferases. These enzymes can catalyze DNA methylation and serve an essential function in chromosome instability and tumor progression [28]. A double knockout of DNMT3A and DNMT3B can strengthen the telomere recombination [29]. In addition, simultaneously silencing both DNMT1 and DNMT3B by using the RNA interference technique has been demonstrated to achieve a synergistic effect in the CpG island methylation in human bladder tumorigenesis [30]. The DNMT3A and 3B genes are located on the chromosomes 2p23.3 and 20q 11.2 and comprise 26exon/25intron and 24exon/23intron, respectively [31]. Recently, public databases have proposed several candidate SNPs in the DNMT3A and 3B genes. Among these SNPs, A→G in the 448 bp upstream of the transcription start site of the promoter region and G→T in the 579 bp from the exon 1B transcription start site have been widely explored. In 2010, Fan et al. used the luciferase assay to prove that the promoter activity of the −448A allele was significantly higher than (more than double) that of the −448G allele, which also increased the risk of gastric cancer [15]. However, most studies have indicated no association between DNMT3A −448A>G and the risk of cancers including endometriosis, gastric atrophy or cancer, and esophageal cancer. [15], [32]–[34]. In addition, the functional effects of DNMT3B −579G>T polymorphisms remain to be elucidated; however, few studies have demonstrated the association between this SNP and the risks of acute myeloid leukemia, Down’s syndrome, immune thrombocytopenic purpura, and colorectal cancer [16], [17], [19], [35]. By contrast, no association has been presented between this SNP and the risks of ovarian cancer, breast cancer, and late-onset Alzheimer’s disease [36]–[38]. Although the exact functional effects of these polymorphisms are not known, we hypothesized that these variants of DNMT3A −448A>G or DNMT3B −579G>T might influence the enzymatic activity of DNMT3A or 3B in DNA methylation. Therefore, in individuals with genetic variants of DNMT3A −448A>G or DNMT3B −579G>T and hereditary or acquired low plasma folate levels (low methyl donor), altered DNA methylation levels might contribute to UC carcinogenesis. In our results, the frequencies of the G and T allelic variants of DNMT3A and DNMT3B in the controls were 80% and 92%, respectively, similar to those reported in previous studies in China and Taiwan [34], [38], [39]; however, no significant association was confirmed between the DNMT3A or DNMT3B polymorphisms and the risk of UC. Nevertheless, in simple logistic models, participants carrying the AG or GG genotypes of DNMT3A or the TT genotypes of DNMT3B exhibited a higher risk of UC compared with those carrying the AA genotypes of DNMT3A or the GG or GT genotypes of DNMT3B, respectively, as indicated by the lower acquired plasma folate levels. Although the results were not statistically significant because of the reduced sample size by stratification, this could probably explain the mechanism of UC carcinogenesis. Future studies with larger sample sizes might confirm our findings and identify the other SNP sites for genotype determination.

Few studies have explored the interaction between the DNMT3A or DNMT3B genotype and plasma folate levels or between the DNMT3A or DNMT3B genotype and cigarette smoking relative to UC risk. Pufulete et al. demonstrated a weak negative connection between plasma folate and colonic DNA hypomethylation [40]. Furthermore, regarding the risks of esophageal squamous cell carcinoma and gastric cardia adenocarcinoma, a significant association was detected between low serum folate levels (<3 ng/ml) and polymorphisms of thymidylate synthase, which also requires 5, 10-methylene-tetrahydrofolate as the methyl donor [23]. In addition, recent studies have indicated that cigarette smoking may modify DNA methylation through the effects of nicotine on the gene expression of DNMT mRNA or DNA-binding factors and then lead to smoking-related diseases [18], [41], [42]. In our study, participants carrying the TT genotypes of DNMT3B and with folate insufficiency or high cumulative cigarette smoking exhibited a 2.3- and 2.6-fold increase in the risk of UC (P<0.05), respectively. Although the present analysis results may not be significant because of the small sample size, our study has the advantage of using an internal dose to measure plasma folate levels.

Several limitations persist when interpreting the present findings. First, we merely measured one single spot level of plasma folate, and thus, the accuracy may be disputable. However, in comparing the differences in plasma folate levels between the incident and prevalent UC cases, we observed that the folate levels were similar for both groups (P = 0.18) and lower than those in the controls; this indicates the reliability of these folate levels under the assumption that all participants had no lifestyle changes. Second, the exact effects of the genetic variants of DNMT3A −448A>G or DNMT3B −579G>T on the expression of their individual enzymatic activities could not be determined. Finally, the sample size of the present study was small, particularly for the analysis of gene polymorphisms and stratification; therefore, the significance of these findings should be interpreted with caution.

In conclusion, this study suggests a significant association between folate insufficiency and a twofold increase in the risk of UC, regardless of the UC patient group. Cigarette smoking was a critical risk factor of UC. No association was observed between the DNMT genotype and the risk of UC. These results suggest that environmental factors played a more crucial role in UC carcinogenesis than did the DNMT3A −448A>G and DNMT3B −579G>T gene polymorphisms. Therefore, large-scale studies using tag-SNPs for genotype determination are recommended, and the association between enzymatic activity and the DNMT3A and 3B genotypes requires confirmation in future studies.
